# Breast biomarkers profile of invasive lobular carcinoma in a cohort of arab women shows no significant differences from carcinoma of no special type

**DOI:** 10.4314/ahs.v22i4.3

**Published:** 2022-12

**Authors:** Areej Al Nemer

**Affiliations:** Associate Professor and consultant, Breast Pathologist, Pathology Department, Imam Abdulrahman Bin Faisal University, Saudi Arabia

**Keywords:** Biomarkers, breast carcinoma types, immunohistochemistry, invasive lobular carcinoma, Ki67

## Abstract

**Objectives:**

Invasive lobular carcinoma (ILC) of the breast is known for its common presentation at an older age, and the frequent expression of favourable profile of estrogen and progesterone receptors (ER & PR) positivity, and human epidermal growth factor receptor 2 (HER2) negativity combined with low proliferation rate as measured by Ki67. This study aimed to test these clinicopathological features of ILC in an Arabic cohort.

**Methods:**

All breast biopsies diagnosed as IDC or ILC were retrospectively reviewed over 2 years period (2017–2018) in an academic centre. Variables were compared using Fisher's exact test for statistical significance.

**Results:**

A total of 134 cases were recruited, 12.7% were ILC. The median age was 52 years for both types. Clustering of ILC cases was noticed in luminal A subtype (47.1%), but there was no statistically significant difference in subtyping between the 2 histologic groups. Ki67 was significantly lower in ILC than IDC category.

**Conclusions:**

Our study showed that ILC in our cohort lacks the advantage of older age and the common high expression of ER positivity in comparison to IDC. There is, however, significant difference of the value of Ki67 proliferation marker. The prognosis of lobular morphology is questionable in our cohort.

## Introduction

Despite the drastic improvement in management, breast cancer (BC) continues to be the most common cancer and one of the leading causes of cancer-related death in women worldwide.^1^ Carcinoma of the breast represents a heterogeneous group of tumor types with diverse morphology, biology and response to therapy. Invasive lobular carcinoma (ILC); first proposed as a distinct entity in 1941 by Foote and Stewart; 2 is the second most common type of BC after invasive carcinoma of no special type or invasive ductal carcinoma (IDC), accounting for 5–15% of all cases.^3^ Besides presenting at an older age, ILC is generally known for being positive forormonal receptors (HR) and negative for human epidermal growth factor receptor 2 (HER2/neu), and having low proliferation rate as measured by Ki67.^4^,^5^ The survival rate and response to systemic treatment of ILC showed variable results in comparison with IDC in different reports.^6^–^9^ In general, BC differs in some features in different races, and it is a disease of a younger age group in Arab countries.^10^ Currently, the management of each case of BC relies mainly on its clinical and pathologic predictive and prognostic factors. Therefore, we performed this retrospective analysis aiming to assess the frequency and the distinction of the clinicopathological characteristics of ILC in our cohort, and to find out whether these features are consistent or variable in different ethnicities.

## Methods

This study is based on the Histopathology Laboratory of an accredited academic center serving a large portion of the society, and it complies with the World Medical Association Declaration of Helsinki. After obtaining the local institutional board review approval, the database was retrospectively reviewed for all cases of breast core needle biopsies (CNB) performed between January 2015-December 2016 and diagnosed as BC. Cases with in situ carcinoma (stage 0), and those with special types other than ILC such as mucinous, papillary, tubular and pure Micropapillary carcinoma were excluded. A total of 134 cases were found eligible for this study, all of them belong to females. For each case, 3 levels of 5 micron-thick hematoxylin and eosin (H&E) stained slides were reviewed by breast pathologist, and confirmation of the diagnoses were performed. As a routine practice, all cases were also stained for biomarkers (estrogen and progesterone receptors (ER & PR), and HER2), along with Ki67 proliferation marker. E-Cadherin was performed as a confirmatory test for all cases of ILC, and to some of the IDC that mimic ILC. No equivocal results were encountered. For all, the intensity of immunohistochemistry (IHC) staining was recorded in percentage, and the cut-off ≥ 1% was accepted as positivity for HR. Because of the lack of consensus for Ki67, the calculations were performed twice using different cut-off points of 14 & 20%. All cases read as 2+ IHC for HER2 were followed by fluorescent in situ hybridization (FISH). Accordingly, the status of HER2 was classified as positive if IHC showed 3+ based on the guidelines, or if IHC was equivocal 2+ but FISH revealed positive gene amplification.^11^

All IHC were performed using Ventana automated slide Stainer, and both positive and negative controls were examined with each run.

Age of the patients, histologic type of BC, the status of ER, PR, HER2, and Ki67 were all collected for the cases. Subtyping based on IHC was performed, and cases were assorted accordingly to luminal A, luminal B, HER2-like, basal like groups. Older age was tested using 2 cut-off points of 40 &50 years.

## Statistical analysis

All variables were compared by Fisher's exact test using GraphPad Prism Software (version 7). Two tailed p value <0.05 was considered to be significant.

## Results

After excluding 15 cases of BC diagnosed as special types other than lobular, 134 total cases were recruited in this study. Seventeen cases were ILC (accounting for12.7% of our cohort, and 11.4 of all invasive BC). All were classic apart from 2 cases of pleomorphic type. The median age for both IDC & ILC was the same (52years), and the age ranges were 26–89 years and 40–69 years for IDC and ILC; respectively. Older age, HR and HER2/neu status failed to show significant statistical difference between the 2 histologic types. Likewise, there was no distinction in subtyping. Among all tested variables, only Ki67 proliferation marker showed significant statistical difference between IDC and ILC using both cut-off points. Table 1 summarizes the tested pathologic parameters of each group.

**Table 1:** Comparison of age and pathologic characteristics between invasive ductal carcinoma and invasive lobular carcinoma of the breast

	IDC, N (%)	ILC, N (%)	p value
Age (years)			
< 40	19 (16.2)	0 (0)	0.129
≥ 40	98 (83.8)	17 (100)	
< 50	52 (44.4)	6 (35.3)	0.603
≥ 50	65 (55.5)	11 (64.7)	
ER			1.000
+ve	83 (70.9)	12 (70.6)	
-ve	34 (29.1)	5 (29.4)	
PR			0.602
+ve	64 (54.7)	11 (64.7)	
-ve	53 (45.3)	6 (35.3)	
HER2/neu			0.119
+ve	28 (23.9)	1 (5.9)	
-ve	89 (76.1)	16 (94.1)	
Ki67			
≤ 14	18 (15.4)	10 (58.8)	0.0003
> 14	99 (84.6)	7 (41.2)	
≤ 20	20 (17.1)	10 (58.8)	0.0005
> 20	97 (82.9)	7 (41.2)	
Luminal A group			
+ve	34 (29.1)	8 (47.1)	0.164
-ve	83 (70.9)	9 (52.3)	
Luminal B group			
+ve	50 (42.7)	5 (29.4)	0.430
-ve	67 (57.3)	12 (70.6)	
HER2 group			0.361
+ve	10 (8.5)	0 (0)	
-ve	107 (91.5)	17 (100)	
Triple negative group			0.748
+ve	23 (19.7)	4 (23.5)	
-ve	94 (80.3)	13 (76.5)	

IDC: invasive ductal carcinoma, ILC: invasive lobular carcinoma, ER: estrogen receptor, PR: progesterone receptor

## Discussion

While the discohesive nature of the monotonous malignant cells is the characteristic feature responsible for poor detection of ILC both on imaging and clinical examination, the classic presentation at an older age coupled with the typical pathologic profile of HR positive, HER2 negative and low Ki67 status are the main factors which confer better prognosis of ILC.^12^–^15^ In this study, we assessed the frequency of these positive factors in our cohort.

The proportion of ILC in our study was similar to the reported up to 15% in the western world^3^, and higher than most Asian series.^16^–^18^ There was no age difference between ILC and IDC in our analysis (Table 1). Likewise, two Korean studies failed to demonstrate a significant association between older age and lobular histology,^19^, ^20^ in contradiction to many others.^1^,^4^,^5^ In general, older age confers better outcome in BC,^21^ and lack of significant age difference in our cohort compromises the advantage of lobular histology in comparison to IDC.

Our results showed 70.6% and 64.7% positivity of ILC cases for ER and PR; respectively. On the other hand, 70.9% and 54.7% of IDC cases were positive for ER and PR; in order. These figures contradict with the reported results by Sastre-Garau et al, which showed more frequent HR positivity in ILC in comparison with IDC.^22^ In general, literature suggests that 80–95% and 70–80% of ER positivity are seen in ILC and IDC; respectively. In contrast, PR positivity was reported to be 60–70% for both types.^23^

HER2 overexpression and amplification is rare in ILC.^3^,^24^,^25^ In the study done by Porter et al, none of the 62 ILC cases studied showed positivity to HER2.25 Our findings of 5.9% of ILC versus 23.9% of IDC positivity to HER2 are comparable to the reported figures by Rakha et al, which showed 4% versus 23% in ILC and IDC; respectively.3 Others also reported HER2 gene amplification or overexpression in 3–5% of ILC.26 Worse prognosis is associated with HER2 positivity as it is considered as independent prognostic factor in BC in general and in ILC in particular.^26^

While the proportion of ER positivity of ILC in our hands is lower than most literature, our results of biomarkers overall are in accordance with the ones reported by Zengel et al of 70.8% ER and 61.4 PR positivity.1 Moreover, they also didn't find a statistically significant difference in HR status between ILC and IDC. In contradiction to our analysis, their study also didn't detect a statistically significant difference in Ki67 expression between the two histologic types using 14 as a cut-off point. The lower Ki67 values of ILC in reference to IDC in our cohort, however, reflect the lower proliferation rate as seen in H&E slides and they match the reported results by others.^4^,^27^

Regarding subtyping, 47.1%, 29.4% and 23.5% of our ILC cases were luminal A, luminal B, and triple negative; respectively. None of our cases belonged to HER2-like group, as the single case which showed HER2 positivity also co-expressed HR's and therefore it was classified as luminal B. While there was more clustering of lobular cases in luminal A group, the difference failed to achieve statistical significance in comparison to the ductal type. Hence, based on our findings there is no statistically significant difference in IHC subtyping between the two histologic types. In agreement with our results, Weigelt et al reported that all molecular subtypes are possibly seen in ILC with predominance of luminal A.^27^

In relation to the significance of subtyping, Engstrom et al reported that luminal A and luminal B ILC cases had similar prognosis, which is worse than luminal IDC.^28^ Adachi et al used different definition of luminal subtyping based solely on ER, and suggested that PR and Ki67 statuses are not associated with the prognosis of luminal ILC.17 Chen et al disagreed with these 2 studies and reported that positivity for both HR is the best in term of outcome and PR negative ILC is the worst among the luminal subtype.^9^ Iorfida et al, on the other hand, reported that each molecular subtype had different outcome in ILC, as they do in IDC.^26^ Thus, the histologic typing of ILC doesn't necessarily carry the same outcome in different cohorts having different biomarker profiles.

Our data suggests that the clinical outcome of ILC in our population is probably less favourable than the international figures, as the main advantage related to the lobular histologic type is the common high expression of ER in comparison to IDC resulting in sparing the patients the morbidity of cytotoxic treatment. Besides, positivity to HR is an undisputed favourable prognostic factor of BC in general. In our studied population, the lack of statistically significant difference in HR status and subsequently the IHC subtypes between ILC and IDC mandates a controlled study of survival and treatment outcome in comparison to the international reported figures. Moreover, ILC is less responsive to chemotherapy than IDC.^6^–^9^ This is most likely attributed to the low Ki67 proliferation marker reported in our results as well as in most international studies making the management plan more challenging for lobular HR negative tumors.

This study was the first attempt to investigate the pathologic features of ILC in an Arab population. We recruited BC cases at first diagnosis by CNB despite the grade, stage and subsequent management plan. Thus, our cohort represents the entire spectrum of cases without selection bias. However, we do have some limitations. First, it is a retrospective study with limited number of cases. Second, it is based in a single institution. A comprehensive multi-centric, nation-based prospective study with adequate follow up period, coupled with gene expression profiling studies are required to consolidate and generalize our results, and to determine the reflection of these pathologic findings on tumor biologic behaviour and prognosis in Arab population.

## Conclusions

Our study showed that ILC in our cohort lacks the advantage of older age and the common high expression of ER positivity in comparison to IDC. There is, however, significant difference of the value of Ki67 proliferation marker. This might suggest that more aggressive treatment options should be considered while counselling our patients. The clinico-pathological features of ILC are not necessarily the same in all ethnicities. This might partially interpret the contradictory results of ILC prognosis in literature.
